# A novel reporting workflow for automated integration of artificial intelligence results into structured radiology reports

**DOI:** 10.1186/s13244-024-01660-5

**Published:** 2024-03-19

**Authors:** Tobias Jorg, Moritz C. Halfmann, Fabian Stoehr, Gordon Arnhold, Annabell Theobald, Peter Mildenberger, Lukas Müller

**Affiliations:** Department of Diagnostic and Interventional Radiology, University Medical Centerof the, Johannes Gutenberg-University Mainz , Langenbeckst. 1, 55131 Mainz, Germany

**Keywords:** Artificial intelligence, Chest X-ray, Radiology workflow, Structured reporting

## Abstract

**Objectives:**

Artificial intelligence (AI) has tremendous potential to help radiologists in daily clinical routine. However, a seamless, standardized, and time-efficient way of integrating AI into the radiology workflow is often lacking. This constrains the full potential of this technology. To address this, we developed a new reporting pipeline that enables automated pre-population of structured reports with results provided by AI tools.

**Methods:**

Findings from a commercially available AI tool for chest X-ray pathology detection were sent to an IHE-MRRT-compliant structured reporting (SR) platform as DICOM SR elements and used to automatically pre-populate a chest X-ray SR template. Pre-populated AI results could be validated, altered, or deleted by radiologists accessing the SR template. We assessed the performance of this newly developed AI to SR pipeline by comparing reporting times and subjective report quality to reports created as free-text and conventional structured reports.

**Results:**

Chest X-ray reports with the new pipeline could be created in significantly less time than free-text reports and conventional structured reports (mean reporting times: 66.8 s vs. 85.6 s and 85.8 s, respectively; both *p* < 0.001). Reports created with the pipeline were rated significantly higher quality on a 5-point Likert scale than free-text reports (*p* < 0.001).

**Conclusion:**

The AI to SR pipeline offers a standardized, time-efficient way to integrate AI-generated findings into the reporting workflow as parts of structured reports and has the potential to improve clinical AI integration and further increase synergy between AI and SR in the future.

**Critical relevance statement:**

With the AI-to-structured reporting pipeline, chest X-ray reports can be created in a standardized, time-efficient, and high-quality manner. The pipeline has the potential to improve AI integration into daily clinical routine, which may facilitate utilization of the benefits of AI to the fullest.

**Key points:**

• A pipeline was developed for automated transfer of AI results into structured reports.

• Pipeline chest X-ray reporting is faster than free-text or conventional structured reports.

• Report quality was also rated higher for reports created with the pipeline.

• The pipeline offers efficient, standardized AI integration into the clinical workflow.

**Graphical Abstract:**

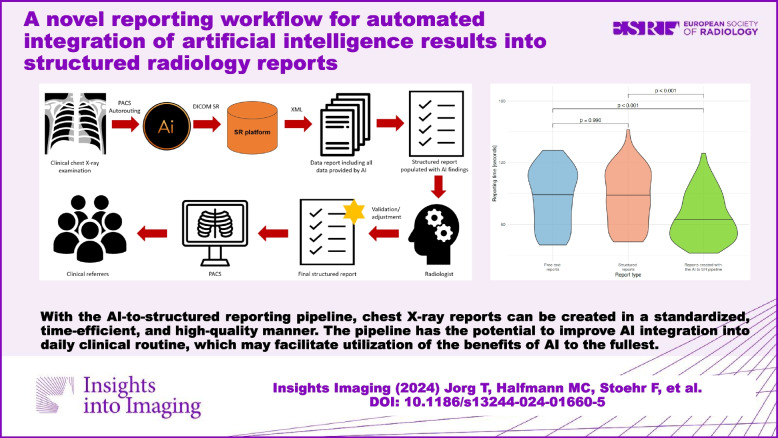

## Introduction

Due to its tremendous potential for helping radiologists facilitate processes in various diagnostic and non-diagnostic tasks, artificial intelligence (AI) is a rapidly evolving field and has gained momentum in radiology in recent years [1–3]. By supporting time-consuming tasks, such as volumetrics, segmentations, or quantifications, AI tools aim to enable improvements in efficiency [2, 3]. In addition, these tools are especially helpful for pathology detection in X-ray and cross-sectional imaging [4–6]. Besides an immense number of scientific publications on the subject, as of December 2023, there are more than 200 commercial CE-marked AI applications for radiology. Moreover, the number of clinical AI users is steadily increasing [7, 8].

For the evidence-based information created by AI to be used diagnostically in clinical routine, the tools need to be properly integrated into radiologists’ workflow. Despite AI integration being a main focus of current research, it is poorly developed, or even completely lacking, at most hospitals, and was recently identified as a main obstacle for clinical AI implementation [7, 9, 10]. Most common AI tools for pathology detection only offer solutions in which their findings have to be reviewed by the radiologist in a separate window external from the reporting workflow [11]. The findings then must be added by the radiologists to their reports manually, off-setting the time-saving potential offered by AI usage. More advanced ways of communicating results are offered by Digital Imaging and Communications in Medicine (DICOM) objects, with which AI findings are automatically sent to the Picture and Archiving System (PACS) [9]. However, this automated transfer of AI results into the PACS also has disadvantages. In addition to the fact that results still have to be manually added to the reports, they are transferred without prior validation from a radiologist. Incorrect AI results (e.g., false positive lung nodules) may be included and made available to every PACS user. This can be misleading, especially for referring physicians who are not trained radiologists and could cause false treatment of patients due to misinterpretation. Lastly, AI results inside the PACS are not automatically transferred to a dedicated structured database from which they could be easily leveraged for datamining purposes such as epidemiologic or scientific research. On the contrary, data collection from AI reports in the PACS would require a great manual effort. A standardized, safe, and time-efficient integration of AI into the radiology workflow is still needed to utilize its full potential and can only be achieved if the transfer of AI-generated findings to radiology reports is automated.

Structured reporting (SR) has been an exciting field of interest in recent years. It first came up as an attempt to structure and standardize radiology reports due to a great heterogeneity in traditional free-text reports in terms of quality, length, and content [12]. Today, SR is often seen as the favourable form of radiology reporting, and its advantages over free-text reporting have been addressed in dozens of studies [13–15]. In addition to improving the quality of the report itself, SR as an IT-based method offers the possibility to aggregate large datasets in a highly structured form, which can be easily leveraged for subsequent data analysis [16–18].

The synergies and dependencies between AI and SR are vast [19]. On the one hand, AI heavily relies on large, structured datasets for training and validation, which are often lacking. Structured data derived from SR has the potential to fill that gap [20, 21]. On the other hand, structured output from AI tools could easily be integrated into structured reports. Moreover, AI in the form of Natural Language Processing (NLP) has already been shown to be helpful for integrating speech recognition into SR [22]. In the future, an even closer entanglement of AI and SR could be useful for improving insufficient clinical AI integration.

Thus, the aim of this study was to exploit this potential and develop a reporting workflow that enables the automated population of SR templates with the results provided by AI tools.

## Methods

### Development of the workflow

At our department, an IHE-MRRT-compliant web-based SR platform, which is fully connected to the radiology information system, was first introduced into clinical routine in 2016 [23]. Since then, reporting templates for various examination types in different imaging modalities have been developed and established in the workflow [24].

AI tools were first implemented into clinical routine in 2018. For development of the new workflow called the AI to SR pipeline, a commercially available tool for automated pathology detection in chest X-rays (Rayscape, Bucharest, Romania) was chosen as a use case. The decision to use chest X-rays was based on the fact that they are one of the most frequent examinations that radiologists have to report on in their daily routine, which means great interest in AI support. It is also an examination of adequate complexity for the development of an SR template.

The AI tool is able to detect multiple lung pathologies, including pneumothorax, pleural effusion, consolidations, opacities, atelectasis, pulmonary oedema, emphysema, cardiomegaly (cardiothoracic ratio), mediastinal/hilar abnormalities, and fractures. Furthermore, it can localize the pathologies in terms of their side and position in the image (left, right, bilateral; lung upper field, middle field, lower field) and to indicate probabilities for the detected pathologies (high, medium, and low).

**Fig. 1:**
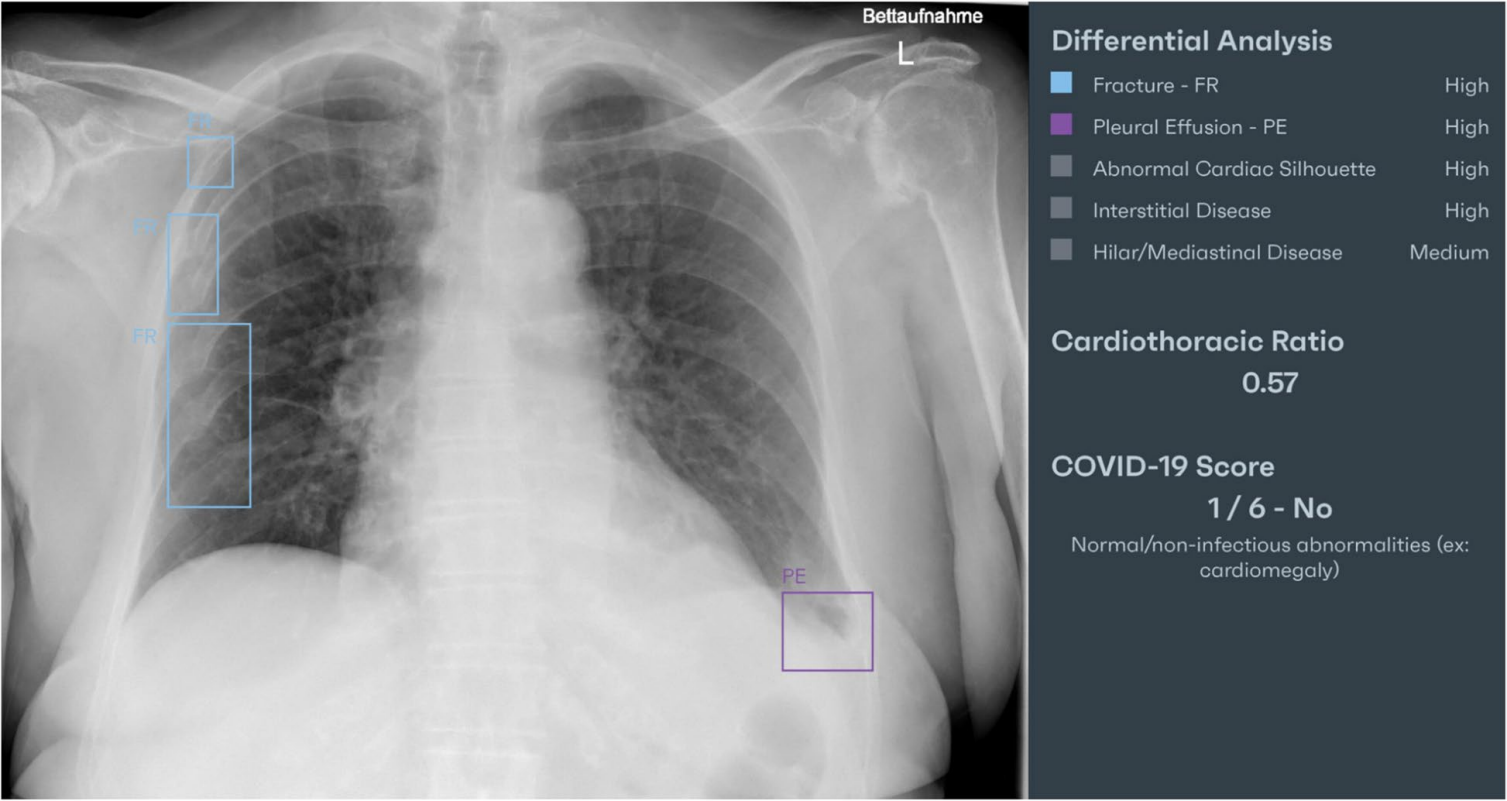
Chest X-ray with marked AI findings

**Fig. 2:**
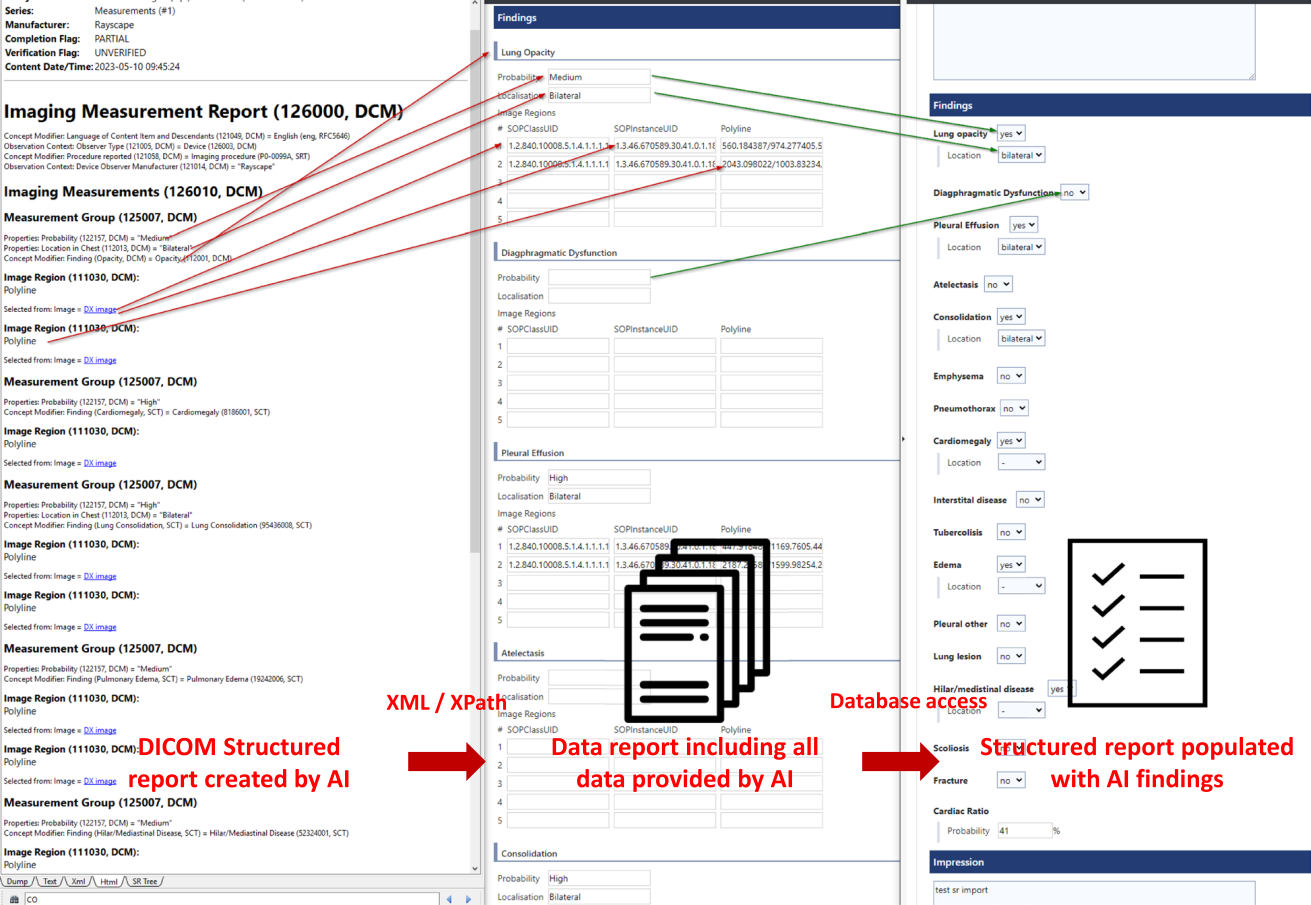
Screenshot of the dataflow from the DICOM SR element generated by AI (left) to the corresponding preliminary data template (middle) and pre-populated SR template (right)

**Fig. 3:**
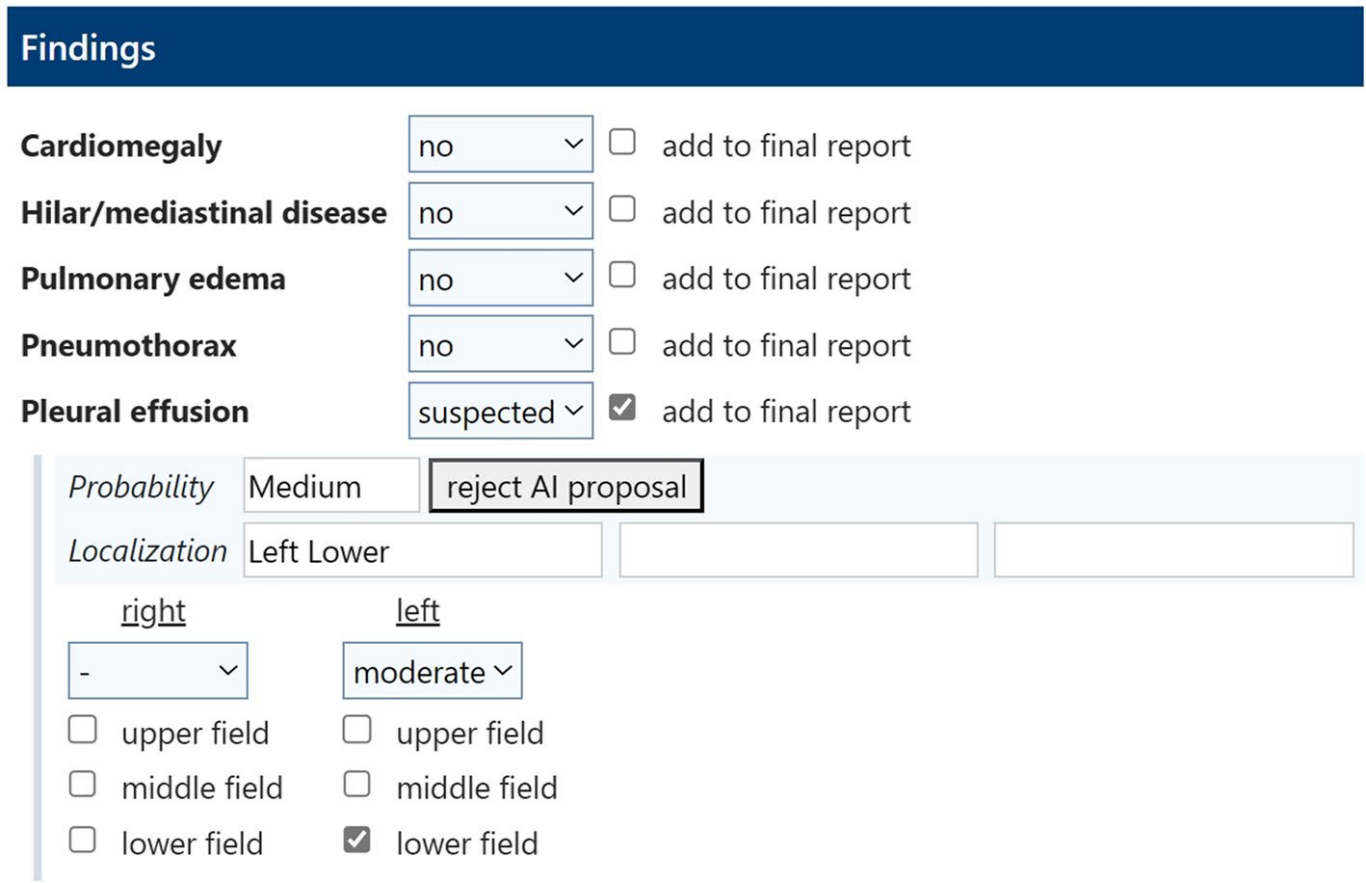
Screenshot of the pre-populated SR template (translated from German to English). In this case, the AI detected a pleural effusion in the left lower field with a medium probability

**Fig. 4:**
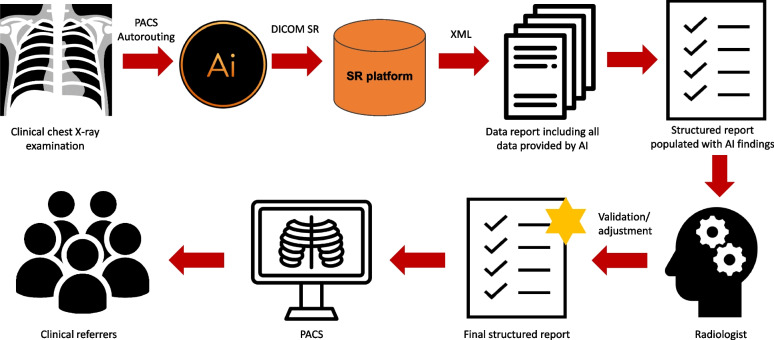
Graphic representation of the full workflow using the AI to SR pipeline

In clinical routine, X-ray images are sent to the PACS after their acquisition. From the PACS, the images are automatically forwarded to the AI for further analysis. Figure 1 is a screenshot of an analyzed X-ray with marked results that are only available to radiologists. For each analyzed chest X-ray, the results are output in DICOM SR format, which takes about 5 min on average from the time the examination is carried out [25]. The DICOM SR object is imported into the SR platform and transformed into XML format. Data from the XML sheet is extracted to a data template via XPath. An intermediate representation in XML offers the advantage that single items from the converted DICOM SR can be directly accessed via XPath. The data template contains all of the AI’s findings and their probabilities, as well as information on their exact coordinates in the matrix of the chest X-ray (polygons/polylines). The exact DICOM image on which the polygons are drawn is referenced via a service object pair (SOP) instance and class unique identifier (UID). From the data template, only clinical findings (including their probabilities and localizations) are extracted to a structured report. Figure 2 is a screenshot of the dataflow. The data transfer takes only a few seconds. The pre-populated structured report is accessed by the radiologist during the reporting process. The radiologist can modify the AI results in the template by changing their location or their probability. Furthermore, the intensities of the findings can be added (e.g., for pleural effusions: slight, moderate, or severe). False AI results can be dismissed from the report by simply clicking on “reject AI finding”. Correct and modified results can be confirmed by clicking “add to final report”. Findings missed by the AI can be added (Fig. 3). After finishing the report, it is released via the radiology information system to the hospital information system and PACS, where it can be accessed by clinical referrers. Figure 4 shows a graphical representation of the full workflow used for the AI to SR pipeline.

In case of unavailability of the platform due to hardware failure or other circumstances, DICOM SR elements created by the AI stay in a queue and are sent once the system is online again. A backup of the database is done daily. DICOM SRs are immediately deleted after conversion while the content of the final structured report is stored in the database. In accordance with the GDPR’s right to be forgotten, all patient data can easily be deleted on request.

### Evaluation of the workflow

The performance of the reporting pipeline was assessed for the chest X-ray tool in two separate ways. As this tool is able to graphically mark its results in the analyzed X-ray in a DICOM secondary capture file (Fig. 1), an evaluation of whether the provided results have been correctly and completely transferred to the structured reports can be made by comparing the marked X-rays with the generated pre-populated reports. This comparison was done for a total of 60 chest X-ray examinations that were retrospectively and randomly chosen from our imaging database. Two board-certified radiologists manually reviewed all AI-analyzed X-rays and corresponding pre-populated structured reports.

In a second approach, three radiology residents created reports for the 60 examinations. Each resident created 20 reports as free-text, 20 reports as conventional structured reports, and 20 reports using the SR to AI pipeline, resulting in a total of 180 reports. AI support was used for all types of reporting, but in the case of free-text and conventional structured reports, the findings provided by AI had to be added to reports manually. To avoid double readings, the study was conducted in a cross-over design (Table 1). For each examination, the radiology residents were shown both the original X-ray image and the secondary capture including graphically marked results provided by the AI. The reporting was done at the beginning of the shift in a normal clinical setting without any particular time pressure. After the completion of the first and second block of 20 reports, there was a break of 30 min. Each resident started the study with a different block (report type). The time to completion was measured for all reports. In addition, two board- and European Diploma in Radiology (EDiR)-certified radiologists defined a gold standard report by consensus reading. The created reports were compared with the gold standard and evaluated by each of the board-certified radiologists on a 5-point Likert scale regarding their completeness and correctness (1 = incorrect/incomplete, 2 = rather incorrect/incomplete, 3 = equivocal, 4 = rather complete/correct, 5 = correct/complete).

**Table 1:** Study design

Radiologist	Free-text reports	Structured reports	AI to SR pipeline
1	Examinations 1–20	Examinations 21–40	Examinations 41–60
2	Examinations 21–40	Examinations 41–60	Examinations 1–20
3	Examinations 41–60	Examinations 1–20	Examinations 21–40

### Statistical analysis

All statistical analyses and graphics were performed in R studio (RStudio Team [2020]. RStudio: Integrated Development for R. RStudio, PBC, http://www.rstudio.com) with R 4.0.3 (A Language and Environment for Statistical Computing, R Foundation for Statistical Computing, http://www.R-project.org). Mean values and standard deviations (SDs), as well as medians and interquartile ranges (IQRs), were calculated over reports. Data distribution was tested using the Shapiro–Wilk test. As the variables were not normally distributed, the non-parametric Mann–Whitney *U* test was used to identify differences. For the assessment of inter-rater agreement, Krippendorff’s alpha was calculated using the following interpretation of the alpha value: 0.0–0.2, slight agreement; 0.2–0.4, fair agreement; 0.4–0.6, moderate agreement; 0.6–0.8, substantial agreement; and 0.8–1.0, near-perfect agreement [26]. *p*-values < 0.05 were considered significant.

## Results

### Study sample

For the 60 included chest X-ray examinations, the median patient age was 69 (IQR 64–77) years and sex distribution 1.1:1 (M:F). All examinations were performed in an anteroposterior chest view; 48% were done in intensive care units, 32% in regular wards, and 20% in the emergency outpatient department. The most common indications for the examinations were post cardiac surgery (25%), suspected pneumonia (23%), decompensated heart failure (20%), central venous catheter position checks (20%), and other (12%).

### Evaluation of the workflow

On the 60 chest X-ray examinations, a total of 294 pathologies were detected by the AI tool. Comparison of the DICOM secondary capture created by the tool with the pre-populated SR template revealed that all of the pathologies were transferred correctly.

**Fig. 5:**
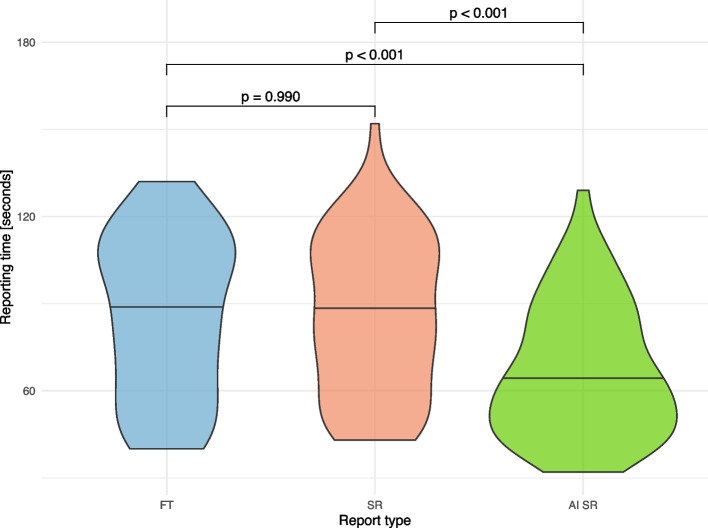
Distribution of the reporting times for the different reporting types. Free-text reports (FT, blue), structured reports (SR, red), and reports created with the AI to SR pipeline (AI SR, green)

The mean reporting time for reports created as free-text was 85.8 s (SD 27.9 s), for structured reports 85.6 s (SD 27.1 s), and for reports using the AI to SR pipeline 66.8 s (SD 23.0 s) (Fig. 5). Though this yielded no significant time difference between free-text and structured reports (*p* = 0.990), the reporting time for AI SR was significantly shorter than either (both *p* < 0.001; Fig. 5).

**Fig. 6:**
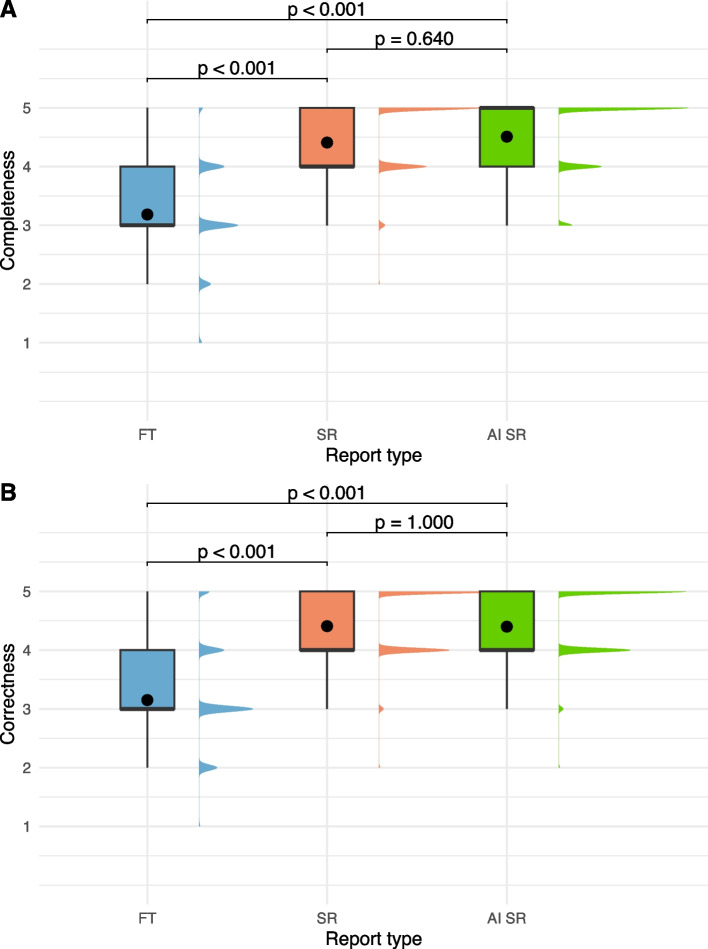
Ratings of the free-text reports (FT, blue), structured reports (SR, red), and reports created with the AI to SR pipeline* (*AI SR, green) regarding completeness (**A**) and correctness (**B**) on a 5-point Likert scale

Regarding the completeness and correctness of the reports, inter-rater agreement was substantial (alpha = 0.73). Mean and median values for the different reporting types regarding completeness and correctness are shown in Table 2 and Fig. 6. Though there were no significant differences between SR and AI SR regarding completeness and correctness of the reports (*p* = 0.640 and *p* = 1.000), both were rated significantly higher than free-text reports (all *p* < 0.001, Fig. 6).

**Table 2:** Completeness and correctness ratings for the different types of reports

Report type	Completeness		Correctness	
	Mean (SD)	Median (IQR)	Mean (SD)	Median (IQR)
Free-text	3.18 (0.85)	3 (3–4)	3.15 (0.80)	3 (3–4)
Structured	4.41 (0.64)	4 (4–5)	4.41 (0.60)	4 (4–5)
AI to SR pipeline	4.51 (0.61)	5 (4–5)	4.40 (0.60)	4 (4–5)

## Discussion

In this study, a new reporting workflow was developed in which SR templates are automatically populated with results provided by AI. The technical feasibility of this AI to SR pipeline was proven by comparing the AI-marked chest X-rays with their corresponding generated structured reports. We found that reports can be generated faster using the AI to SR pipeline compared with free-text reporting and conventional SR. In addition, subjective quality assessment revealed higher ratings for reports created with the pipeline compared to free-text reporting.

AI tools have been shown to be able to help radiologists in evaluating models for dozens of examination types [1–6]. However, in a real clinical setting, the impact of AI on the reading times for radiologists is unclear. A recent prospective study showed that AI abnormality detection on chest X-rays had only a marginal impact on time savings and, in complex cases, may even lead to longer reading times than without the AI support [27]. In the present study, we were able to show that AI-aided reporting times for chest X-rays are dependent on the type of reporting used and is quicker if automated transfer of AI results into structured reports is applied. This underlines the fact that a standardized, seamless, and time-efficient integration of AI is strongly needed in order to take full advantage of its potential in clinical routine.

In addition, reports are generated as structured reports and automatically stored in a database, enabling subsequent data analysis. This is not only advantageous for radiologists who can use the gained data for research purposes and quality assurance but could also be of use for the corresponding AI tool in the future. The possible creation of a feedback loop in which the user-validated results are returned to the AI tools in a highly structured form would enable continuous learning of the AI and further improve its performance [28]. Moreover, validated AI results that are released to referring physicians in the form of a structured report do not carry the risk of misinterpretation as with unvalidated secondary capture results in the PACS.

AI-based radiology workflow optimization is currently a major topic in the scientific community, with lots of research being conducted. Most of the published work has focused on optimization of components of the workflow other than report generation (e.g., AI-aided improvements in scan protocols, work lists, or hanging protocols) [1, 29, 30]. The studies that do focus on the reporting process include NLP-based integration of speech recognition into SR, large language model (LLM)-based creation of structured reports from free-text reports, or the automated prediction of the impressions section of free-text reports [22, 31, 32]. A very recent study described the possibility of AI generating structured reports for pulmonary embolism CTs in order to enable continuous AI learning [28]. However, the clinical applicability of this approach has not yet been investigated in a user study with radiologists. To the best of our knowledge, the approach presented here is the first clinically deployed system to automatically integrate AI results into structured radiology reports.

Some commercial AI pathology detection tools are able to automatically create free-text reports of their findings. However, these have to be manually transferred to the official radiology report by the radiologists because there is no workflow integration. In addition, they lack structure because they are free-form.

For this study, we showed that an AI to SR reporting pipeline was successfully established for the use case of an AI tool for chest X-rays. In general, the pipeline could easily be adopted for any other AI tools, provided they are capable of creating a DICOM SR file of the results. We are currently working on extending the applicability of the reporting pipeline to more tools, including self-developed tools (e.g., automated spleen and kidney segmentation, sarcopenia quantification for abdominal CT) and commercially available tools (e.g., lung nodule detection and pattern recognition for chest CT). However, for the time being, each AI tool has to be separately integrated into the SR platform and a customized SR template created, which requires great effort. In the future, with emerging app store-like AI platforms from commercial vendors, a single integration of the entire platform could replace the need for connections between the SR platform and individual AI applications. Moreover, successful clinical implementation of AI platform solutions is strongly dependent on seamless workflow integration, which requires few, if any, extra interactions by radiologists. Commercial SR platforms could offer additional advantages here, particularly due to their high interoperability standards, their widespread use and availability of large amounts of customizable templates [33].

This study has several limitations. First, the performance analysis of the pipeline was performed at a single site in a retrospective design with no external validation. Second, SR is not the main form of radiology reporting despite its advantages and an extensive amount of research [34, 35]; it is not equally applicable to all types of radiological examinations, which will also be the case for the AI to SR pipeline. In the case of chest X-rays, findings are not always as clear as they are in cross-sectional imaging, and even experienced radiologists may disagree on image interpretation. As structured reports leave little room for vague wording, radiologists tend to refrain from using them for chest X-rays. However, an SR-based integration of AI into the workflow can only be successful if SR itself will succeed. In the future, the possibility of creating high-quality AI-aided structured reports in less time than free-text reports together with an improved conversion of free-text into structured reports fostered by NLP and LLMs may lead to increased acceptance of SR among radiologists.

We used DICOM SR and XML standards for the technical development of the pipeline. However, in the future, newer standards including FHIR might be more suitable once they are established and widely available.

Lastly, since the primary aim of the study was to evaluate whether the new reporting pipeline can improve AI integration, we did not focus on the performance of AI. The ratio of false positive or false negative AI findings was not calculated, but is still of great interest and should be addressed in future studies.

## Conclusion

We successfully developed a new reporting workflow capable of automatically integrating findings provided by AI tools into structured reports. With this so-called AI to SR pipeline, chest X-ray reports can be created in a standardized, time-efficient, and high-quality manner. Thus, the pipeline has the potential to improve AI integration, which is strongly needed in order to utilize the full possibilities offered by AI support in daily clinical routine. It can particularly help enhance synergies between AI and SR.

## Data Availability

The datasets used for this study are available from the corresponding author on reasonable request.

## Abbreviations

AI: Artificial intelligence
DICOM SR: Digital Imaging and Communications in Medicine Structured Report
DICOM: Digital Imaging and Communications in Medicine
IHE MRRT: Integrating the Healthcare Enterprise Management of Radiology Report Template
LLM: Large language model
NLP: Natural language processing
PACS: Picture and archiving system
SR: Structured reporting
